# The effects of prolonged prone positioning on response and prognosis in patients with acute respiratory distress syndrome: a retrospective cohort study

**DOI:** 10.1186/s40560-025-00795-x

**Published:** 2025-05-07

**Authors:** Yuhang Yan, Junying Bao, Shumin Cai, Xiangning Zhong, Bingxuan Geng, Jingyi Liang, Zhiya Deng, Zhongqing Chen, Zaisheng Qin, HongBin Hu, Zhenhua Zeng

**Affiliations:** 1 Department of Critical Care Medicine, Nanfang Hospital, Southern Medical University, Guangzhou, 510515 China; 2 School of Nursing, Southern Medical University, Guangzhou, 510515 China; 3 Department of Anesthesiology, Nanfang Hospital, Southern Medical University, Guangzhou, 510515 China

**Keywords:** Prolonged prone position, Acute respiratory distress syndrome, Response rates, Intensive care unit, Prognosis

## Abstract

**Background:**

Prone positioning improves outcomes in patients with acute respiratory distress syndrome (ARDS), but the optimal duration in critical care settings remains uncertain. This study aims to evaluate the investigates the impact of prone ventilation duration on clinical outcomes.

**Methods:**

This retrospective study was conducted on ARDS patients admitted to the intensive care unit (ICU), Nanfang hospital of Southern Medical University, who received prone positioning. Patients were categorized into two groups: the prolonged prone positioning (PPP) (≥ 16 h) group and the standard prone positioning (SPP) (< 16 h) group. Propensity score matching (PSM) was employed to balance baseline characteristics. Cox proportional hazards, regression models were utilized to evaluate the association between the prone duration and clinical outcomes. Kaplan–Meier survival curves were generated to compare 28-day mortality, with log-rank tests analyzing differences. Restricted cubic spline (RCS) were applied to investigate the time–response between prone duration, PaCO₂, PaO₂, positive end-expiratory pressure, response rate, and 28-day mortality. In addition, the incidence of prone position-related complications was assessed in both groups.

**Results:**

A total of 234 patients with ARDS were included, with an overall 28-day mortality of 49.1% (115/234). After PSM, 81 matched pairs were compared. The PPP group had lower 28-day mortality (46.9% vs. 53.1%; hazard ratios (HR): 0.53; 95% CI 0.32–0.85; *P* = 0.033) and improved prone positioning response rate [70.5% vs. 60.5%; odds ratio (OR): 1.46; 95% CI 1.23–1.89; *P* = 0.025]. RCS analysis suggested a reduction in mortality with prone durations ≥ 16 h, and longer durations correlated with better prone response. However, no significant association was found between PPP and reduced ICU or hospital length of stay. RCS analysis indicated a gradual decrease in 28-day mortality with increasing duration of prone positioning, and longer duration were associated with a higher likelihood of a prone response. There were no significant differences in prone ventilation-related complications between the two groups.

**Conclusions:**

PPP (≥ 16 h) is associated with reduced 28-day mortality and improved response rates in ICU patients with ARDS, without increasing complication risks. Prospective studies are needed to further validate these results.

**Supplementary Information:**

The online version contains supplementary material available at 10.1186/s40560-025-00795-x.

## Background

Acute respiratory distress syndrome (ARDS) is a severe respiratory condition characterized by progressive respiratory distress and refractory hypoxemia, resulting from acute, diffuse, inflammatory lung injury and increased pulmonary capillary permeability that leads to pulmonary edema [1]. It poses a significant burden on intensive care unit (ICU), with observational studies showing that ARDS accounts for 10.4% of ICU admissions, accompanied by high mortality rates, with mortality rates exceeding 40% in moderate to severe cases [2].

Current treatment strategies for ARDS primarily focus on etiological treatment, respiratory support, and anti-infective therapy, with limited efficacy in reducing mortality [3]. Prone positioning ventilation (PPV), first reported in 1970, has been recognized as an effective method for improving oxygenation and facilitating lung recruitment in acute respiratory failure [4]. The potential benefits include improved ventilation/perfusion ratios, increased end-expiratory lung volume, and better tidal volume distribution, contributing to a reduction in ventilator-induced lung injury (VILI) [5, 6].

However, PPV is a resource-intensive intervention requiring skilled personnel and increasing nursing workload [7]. It may also lead to hemodynamic instability and possible VILI exacerbation through atelectrauma caused by frequent turning [8–10]. Real-world studies have shown considerable variation in the duration of PPV, and the optimal duration remains uncertain. A meta-analysis suggests that a minimum of 12 h of PPV per day significantly reduces mortality in severe ARDS patients [11]. Recent studies have indicated that extending PPV beyond 24 h, particularly in COVID-19 patients, has shown further improvements in oxygenation and outcome [12]. Current guidelines also recommend utilizing PPV for 12–16 h daily, alternating with 8 h of supine positioning [13–15], but recent update of the ESICM guidelines for ARDS management still lack specific guidance on optimal duration [16]. Current studies referenced in these guidelines primarily focus on comparing the outcomes of prone vs. supine positioning, leaving the effects of extended prone time understudied. Recent research has introduced the concept of prone positioning response, defined as an increase of ≥ 20 mmHg in PaO_2_/FiO_2_ ratio following prone positioning, to evaluate efficacy [17, 18]. While beneficial in assessing patient response with this concept, its relationship with prognosis and optimal PPV duration remains unclear [17, 19, 20]. As Cinnella.et al. highlighted [20], distinguishing between responders and non-responders poses a key challenge. Although prolonged prone positioning may offer certain benefits to the patients, evidence regarding its impact on outcomes is still insufficient, and the relationship between patient response and prognosis remains unclear.

This study aims to explore the effects of prolonged PPV duration on ARDS patients’ response and outcomes. By examining the relationship between prone duration, patient response, and prognosis, this research seeks to optimize PPV strategies, improve patient outcomes, and reduce healthcare resource utilization.

## Methods

### Study design

This is a single-center, retrospective cohort study that included patients admitted to the ICU of Nanfang Hospital, Southern Medical University in China from December 2022 to December 2023. Ethical approval was obtained from the Ethics Committee of Nanfang Hospital, Southern Medical University (approval number: NFEC-202312-K61). The study adhered to the principles of the Helsinki Declaration, and patient information was de-identified prior to analysis in accordance with its criteria.

### Population

This study included patients diagnosed with ARDS based on the Berlin definition [1], who underwent at least one session of prone position ventilation therapy. Patients with an ICU stay of less than 24 h were excluded, as were those whose initial prone position ventilation session lasted less than 6 h [21]. Clinical treatments for ARDS patients, including respiratory management and medication therapy, adhered to the ARDS guidelines [1, 15].

### Data collection

Data were systematically collected on demographics, disease etiology, arterial blood gas analysis and laboratory results from the hospital's medical records system during each patient's ICU admission. We also recorded the sequential organ failure assessment (SOFA) score, acute physiology and chronic health evaluation II (APACHE II) score, and Murray score to assess the severity of illness. The use of extracorporeal membrane oxygenation (ECMO) and muscle relaxants, as well as the duration of mechanical ventilation during the ICU stay and the occurrence of re-intubation in patients were recorded. Information on prone position ventilation therapy, including initiation and cessation times, total treatment duration, session frequency, and days on which prone was performed were documented. In addition, the occurrence of prone positioning-related complications was collected from the nursing records in the ICU. Arterial blood gas analysis was performed 2 h before and after the first prone session to obtain the PaO2/FiO2 ratio. Mechanical ventilation parameters before and after prone positioning, such as positive end-expiratory pressure (PEEP), compliance, tidal volume, FiO_2_, and etCO_2_, have also been systematically collected. A 28-day follow-up was conducted post-initial prone position ventilation to determine patient survival status, either through a review of medical records or telephone interviews.

### Group and outcomes

Patients were divided into two groups based on the duration of their first prone session: ≥ 16 h (prolonged prone positioning, PPP) or < 16 h (standard prone positioning, SPP) [11, 13, 22]. Patients who demonstrated an improvement of ≥ 20 mmHg in the PaO_2_/FiO_2_ ratio after the initial prone ventilation will be considered responders, while non-responders did not achieve this improvement [20, 23]. The 28-day mortality and response rates were the primary outcomes. Secondary outcomes included the duration of mechanical ventilation during the ICU stay, the ICU reintubation rate, ICU mortality and the length of ICU and hospital stays.

Regarding safety outcomes, based on the management guidelines for ARDS and expert consensus on prone ventilation both domestically and internationally [11, 16, 24, 25], the following prone positioning-related complications were collected: (1) catheter-related events: displacement or dislodgement of central venous catheters, accidental extubation of the endotracheal tube; (2) hemodynamic instability: defined as a decrease in mean arterial pressure > 20% during prone positioning or an increase in the dose of vasoactive medications ≥ 50%; (3) pressure injuries: skin damage at bony prominences (e.g., face, shoulders, and ankles) assessed according to the NPUAP staging criteria [26]; (4) gastrointestinal complications: including intolerance to enteral nutrition, nausea, vomiting, etc.; and (5) other complications: including optic nerve damage due to periorbital edema, brachial plexus neuropathy, and others.

### Statistical analysis

We determined the sample size based on both the mortality rate and response rate. According to the latest research on prone positioning, the relative risk (RR) of PPP compared to SPP is 0.477 [12]. The 28-day mortality rate for ARDS patients receiving prone ventilation ranges from 35 to 60% [12, 18, 27, 28]. Assuming a mortality rate of 35%, with a significance level of *α* = 0.05 and a power of 1-*β* = 0.80, the sample size calculation indicates that at least 87 patients per group are required. Regarding the response rate, the prone positioning response rate for ARDS patients ranges from 50 to 70% [18, 27]. We anticipate that the response rate for the PPP group will be 70%, and for the SPP group, it will be 50%. With a significance level of *α* = 0.05 and a power of 1-*β* = 0.80, the sample size calculation indicates that at least 91 patients per group are required. As a retrospective study, we collected patient data to the best of our ability. After completing the case collection, we conducted a post hoc sample size calculation, which confirmed that our sample size met the requirements for the two primary endpoints.

All statistical analyses and visualizations were performed using R software version 4.2.2. Variables with more than 10% missing data were excluded from the study. For variables with 5–10% missing data, multiple imputation was applied, while those with less than 5% missing data were imputed using the mean value. Continuous variables were expressed as medians with interquartile ranges (IQR) and compared using the Mann–Whitney *U* test. Categorical variables were presented as counts and percentages, with comparisons performed using chi-square or Fisher’s exact tests.

Propensity score matching (PSM) was performed with a caliper of 0.25, ensuring a 1:1 ratio to balance baseline characteristics between groups. Matching results were evaluated with standardized mean difference (SMD), jitter plots, and common support domain plots (Table S1, Figures S1–3).

Cox regression models were employed to identify independent prognostic factors for ARDS patients. Logistic regression analysis was conducted to assess the effect of prone positioning duration on patients’ response, and its impact on prognosis. Linear regression was performed to evaluate the correlation between PPP and both ICU length of stay and total hospital stay. Kaplan–Meier survival curves illustrated survival differences analyzed using log-rank tests. Stratification analysis was conducted to determine whether the association between prone duration and 28-day mortality varied across subgroups. Restricted cubic splines (RCS) were generated to explore the connections among factors, prone positioning response, and patient prognosis. All statistical tests were two-sided, with a *P* < 0.05 deemed statistically significant.

## Results

**Fig. 1:**
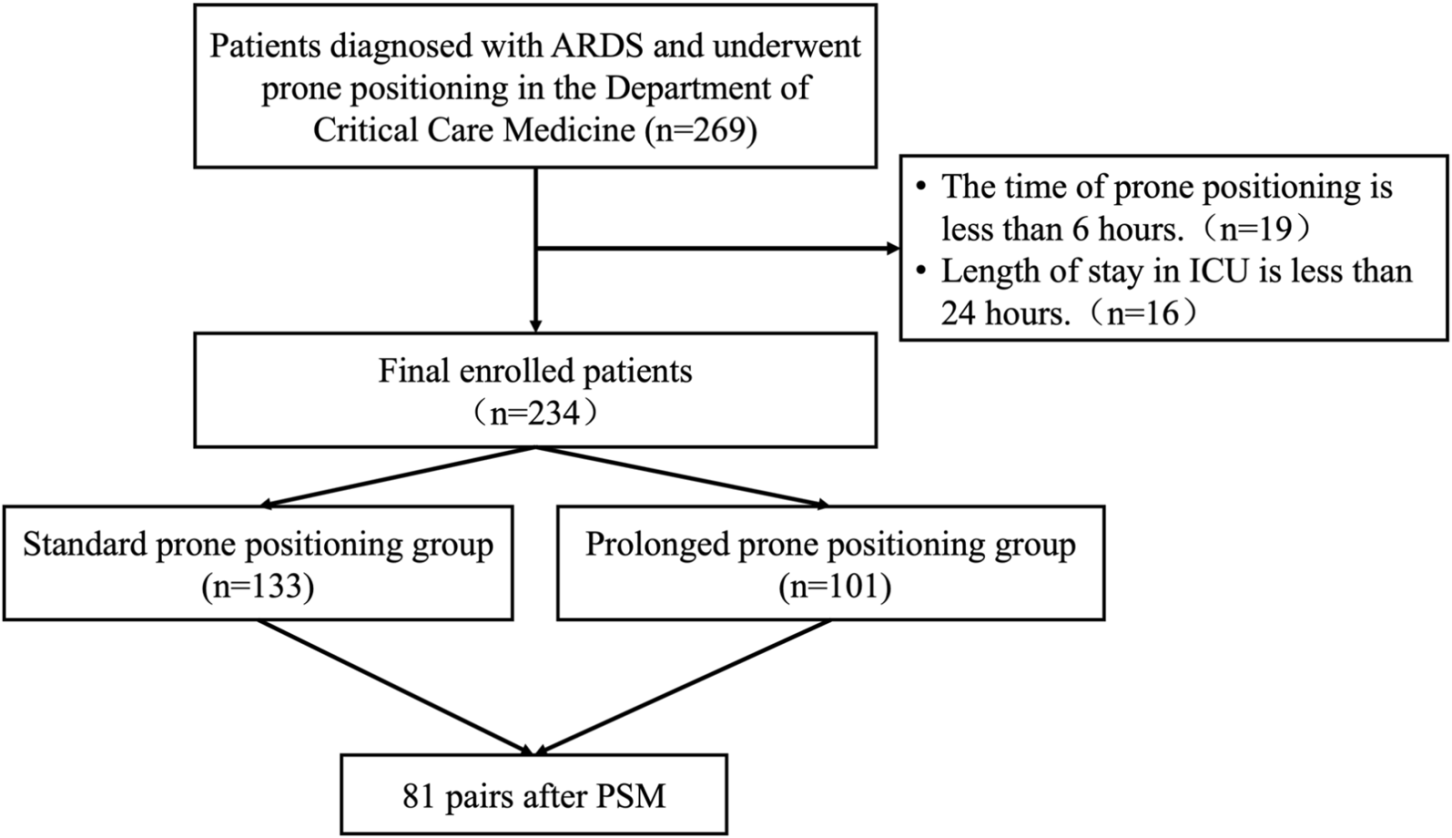
Enrollment process of ARDS patients underwent prone positioning. *ARDS* acute respiratory distress syndrome, *ICU* intensive care unit, *PSM* propensity score matching

During the study period, a total of 269 critically ill patients were admitted with ARDS and underwent prone positioning (Fig. 1). After applying the exclusion criteria, 234 patients were included in the analysis; 101 (43.2%) patients received prolonged prone position. One hundred thirty-three (56.8%) patients received standard prone position. As shown in Table 1, there were significant differences in the age, gender, etiologies and microbiological profiles between the PPP and SPP groups. The use of mechanical ventilation (MV) was more common in the PPP group (96.0% vs. 88.7% *P* = 0.045). The overall use of muscle relaxants in patients was 39.3%, with no statistical difference observed between the two groups. No significant differences were observed in the mechanical ventilation parameters between the two groups. The SOFA score, APACHE II and Murray score were similar between the groups. The number of the prone position sessions during ICU stay was 2 in the PPP group and 3 in the SPP group, and the difference was not statistically significant (*P* = 0.078). No significant differences were observed in the mechanical ventilation parameters before and after proning. Further prone positioning information and respiratory parameters before and after proning are detailed in Table S2. According to the Berlin definition [1], 200 patients (85.5%) were classified as having moderate to severe ARDS.

**Table 1:** Baseline characteristics between two groups before PSM

Items	Overall (*n* = 234)	SPP group (*n* = 133)	PPP group (*n* = 101)	*P*
Age	63.00 [52.00, 73.00]	61.00 [51.00, 72.00]	65.00 [55.00, 76.00]	0.033
Gender [*n*(%)]				
Male	186 (79.5)	103 (77.4)	83 (82.2)	0.029
Female	48 (20.5)	30 (22.6)	18 (17.8)	
Height (m)	160.0 [155.0, 170.0]	158 [154.0, 171.0]	160.0 [155.0, 170.0]	0.391
Weight (kg)	60.0 [52.5, 66.6]	59.9 [50.1, 66.2]	60.5 [51.5, 65.6]	0.091
Body mass index (kg/m^2^)	22.8 [21.9, 24.8]	22.5 [21.1, 24.5]	23.0 [21.3, 25.0]	0.439
Etiologies [*n*(%)]				
Pneumonia	226 (96.6)	130 (97.7)	96 (95.0)	0.447
Sepsis	82 (35.0)	41 (30.8)	41 (40.6)	0.058
Trauma	14 ( 6.0)	4 (3.0)	10 (9.9)	0.054
Other etiologies	25 (10.7)	9 (6.8)	16 (15.8)	0.044
ARDS severity [*n*(%)]				
Mild	34 (14.5)	19 (14.3)	15 (14.9)	0.911
Moderate	138 (59.0)	80 (60.2)	58 (57.4)	
Severe	62 (26.5)	34 (25.6)	28 (27.7)	
Microbiology				
Virus [*n*(%)]	193 (82.5)	116 (87.2)	77 (76.2)	0.044
Bacteria [*n*(%)]	177 (75.6)	99 (74.4)	78 (77.2)	0.735
Fungus [*n*(%)]	109 (46.6)	62 (46.6)	47 (46.5)	1
APACHE II	23 [18, 29]	23 [20, 28]	23 [16, 31]	0.779
SOFA score	8 [5, 9]	8 [5, 10]	9 [5, 10]	0.273
Murrary score	3.00 [2.50, 3.50]	3.00 [2.00, 3.50]	3.00 [2.50, 3.50]	0.824
Charlson index	5 [4, 7]	5 [4, 7]	6 [4, 7]	0.369
Number of the prone position sessions	2 [1, 5]	3 [1, 5]	2 [2, 5]	0.078
Days from ICU to pronation (days)	1.67 [0.74, 4.70]	1.75 [0.82, 4.95]	1.44 [0.69, 4.01]	0.052
Treatment [*n*(%)]				
Mechanical Ventilation	215 (91.9)	118 (88.7)	97 (96.0)	0.045
Muscle relaxants	92 (39.3)	51 (38.4)	41 (40.6)	0.124
ECMO	28 (12.0)	16 (12.0)	12 (11.9)	0.087
Mechanical ventilation parameters				
PEEP (cmH_2_O)	10 [8, 10]	10 [8, 11]	10 [8, 10]	0.703
Compliance (mL/cm H_2_O)	32 [23, 40]	32 [22, 40]	32 [24, 39]	0.773
etCO_2_ (mmHg)	35 [33, 47]	37 [33, 46]	36 [34, 46]	0.182
FiO_2_ (%)	70 [50, 100]	70 [50, 100]	70 [55, 100]	0.711
Tidal volume (mL/kg)	7.3 [6.0, 9.0]	7.1 [5.9,9.1]	7.4 [6.1,9.0]	0.314
Arterial blood gas measurements				
PaO_2_ (mmHg)	93.05 [77.00, 125.50]	94.50 [78.70, 127.00]	91.80 [75.20, 121.00]	0.271
PaCO_2_ (mmHg)	42.30 [37.60, 52.10]	42.20 [37.60, 48.70]	43.20 [37.53, 55.22]	0.474
PaO_2_/FiO_2_ ratio	145.00 [100.25, 203.75]	149.00 [101.00, 213.00]	145.00 [97.00, 200.00]	0.360

*PSM* propensity score matching, *ARDS* acute respiratory distress syndrome, *APACHE II* acute physiology and chronic health evaluation II, *SOFA* sequential organ failure assessment, *ICU* intensive care unit, *ECMO* extracorporeal membrane oxygenation, *PEEP* positive end-expiratory pressure

**Fig. 2:**
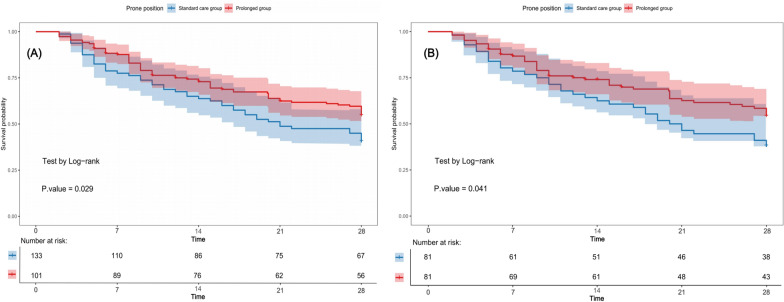
Kaplan–Meier curves of the PPP group and the SPP group before and after propensity score matching. **A** Kaplan–Meier curve for 28-day mortality according to prone duration before propensity score matching. **B** Kaplan–Meier curve for 28-day mortality according to prone duration after propensity score matching

Logistic regression analysis was conducted to examine the impact of the duration of prone positioning on the response state. PPP was found to be significantly associated with increase rate of positive response (72.3% vs. 60.9%; OR, 1.67; *P* = 0.023). The Cox proportional hazards model was used to examine the differences in mortality outcomes between the two groups. In the pre-matched cohort, PPP was associated with reduced 28-day mortality (45.5% vs. 50.4%; Hazard Rate, HR, 0.74; *P* = 0.038) after adjusting for potential confounding factors. After PSM, 81 matched pairs of PPP and SPP patients were identified. Consistent with pre-matched results, PPP was associated with reduced 28-day mortality (46.9% vs. 53.1%, HR, 0.53; 95% CI 0.32–0.85; *P* = 0.033) and an increased response rate (70.5% vs. 60.5%; OR, 1.46; 95% CI 1.23–1.89; *P* = 0.025). Log-rank tests confirmed statistically significant differences in 28-day mortality between the two groups, as shown in Fig. 2. However, no significant association was found between PPP and reduced ICU or hospital length of stay before and after PSM (Table 2).

**Table 2:** Association between prone duration and clinical outcomes in critically ill patients with ARDS before and after PSM

	Overall	SPP group	PPP group	*P*	HR/OR(95% CI)
Before PSM	*n* = 234	*n* = 133	*n* = 101		
Response rate^a^	154 (65.8)	81 (60.9)	73 (72.3)	0.02	1.67 (1.16–2.95)
Length of MV (days)^b^	6.26 [2.77, 13.42]	6.40 [2.56, 12.62]	6.17 [3.24, 15.00]	0.64	−0.12 (−0.99 to 1.57)
Length of ICU stay (days)^b^	13.00 [7.08, 22.00]	14.00 [8.00, 25.00]	14.00 [6.00, 21.00]	0.56	−1.33 (−5.83 to 3.16)
Length of hospital stay (days)^b^	19.00 [10.00, 24.00]	20.00 [11.00, 25.00]	19.00 [9.00, 24.00]	0.41	−1.03 (−3.53 to 1.47)
Re-intubation in ICU, *n*(%)^c^	64 (29.8)	36 (30.5)	28 (28.9)	0.99	0.95 (0.02–1.96)
ICU mortality, *n*(%)^d^	102 (43.6)	60 (45.1)	42 (41.6)	0.06	0.88 (0.42–1.14)
28-day mortality^d^	113 (48.3)	67 (50.4)	46 (45.5)	0.04	0.74 (0.57–0.92)
After PSM	*n* = 162	*n* = 81	*n* = 81		
Response rate^a^	106 (65.4)	49 (60.5)	57 (70.4)	0.03	1.46 (1.23–1.89)
Length of MV (days)^b^	7.07 [2.85, 13.68]	7.15 [2.99, 14.00]	6.89 [2.75, 13.61]	0.79	−0.42 (−2.28 to 2.01)
Length of ICU stay (days)^b^	13.50 [7.00, 22.32]	14.00 [7.53, 23.00]	13.00 [7.00, 21.00]	0.39	−1.91 (−7.06 to 3.24)
Length of hospital stay (days)^b^	20.00 [10.00, 23.00]	19.00 [10.00, 24.00]	19.00 [9.00, 24.00]	0.78	−0.68 (−3.65 to 2.32)
Re-intubation in ICU, n(%)^c^	45 (29.8)	22 (29.7)	23 (29.9)	0.86	1.03 (0.22–2.28)
ICU mortality, *n*(%)^d^	76 (46.9)	39 (48.1)	37 (45.7)	0.07	0.70 (0.41–1.21)
28-day mortality^d^	81 (50.0)	43 (53.1)	38 (46.9)	0.03	0.53 (0.32–0.85)

*ARDS* acute respiratory distress syndrome, *PSM* propensity score matching, *MV* mechanical ventilation, *ICU* intensive care unit, *HR* hazard ratio, *OR* odds ratio

^a^Response is defined as an increase in PaO_2_/FiO_2_ ratio by more than 20 mmHg after prone positioning. Logistic regression was used to assess the effect of prone positioning duration on the response

^b^Linear regression was performed to evaluate the correlation between prolonged prone positioning and both ICU length of stay and total hospital stay. HR was calculated using the formula HR = e βi. This section includes only patients who received mechanical ventilation

^c^Logistic regression was used to evaluate the relationship between prone positioning duration and the reintubation rate

^d^Cox regression was used to assess the impact of prone positioning duration on ICU and 28-day mortality outcomes. The adjusted variables included those with a *P* < 0.05 in univariate analysis, as well as confounders selected based on the judgment of experienced clinicians. These variables included age, APACHE II, SOFA, Murray score, prone positioning duration, response status, days on mechanical ventilation, and the time from ICU admission to the start of prone

In this study, a total of 123 patient were collected data of prone positioning-related complications. The overall incidence of complications was 49.0% (60/123), compared with 50% and 47.9% in the PPP group and SPP group, with no statistically significant difference. The most common complication was pressure injury, which had an incidence rate of 38.2%. No significant differences were observed between the PPP group and SPP group in terms of catheter-related events, hemodynamic instability, gastrointestinal-related complications, or other complications (Table 3). Information regarding the missing data is presented in Supplementary Material Table S3, with no significant difference between two groups. The sensitivity analyses using best/worst-case imputation and multiple imputation methods for missing data also are shown in Tables S4 and S5. As for effect evaluation, the overlap between the curves before and after imputation is good, demonstrating the effectiveness of the imputation (Figure S4).

**Table 3:** Safety analysis of prolonged prone position ventilation for patients with prone positioning-related complications

Prone positioning-related complications	Overall (*n* = 123)	SPP group (*n* = 73)	PPP group (*n* = 50)	*P*
Any complication of proning, *n*(%)	60 (49.0)	35 (47.9)	25 (50.0)	0.078
Catheter-related events, *n*(%)	3 (2.4)	2 (2.7)	1 (2.0)	0.912
Hemodynamic instability, *n*(%)	13 (10.6)	7 (9.6)	5 (10.0)	0.831
Pressure injury, *n*(%)	47 (38.2)	27 (37.0)	20 (40.0)	0.138
Gastrointestinal complications, *n*(%)	17 (13.9)	10 (13.7)	7 (14.0)	0.634
Other complications, *n*(%)	13 (10.7)	8 (11.0)	5 (10.0)	0.214

**Fig. 3:**
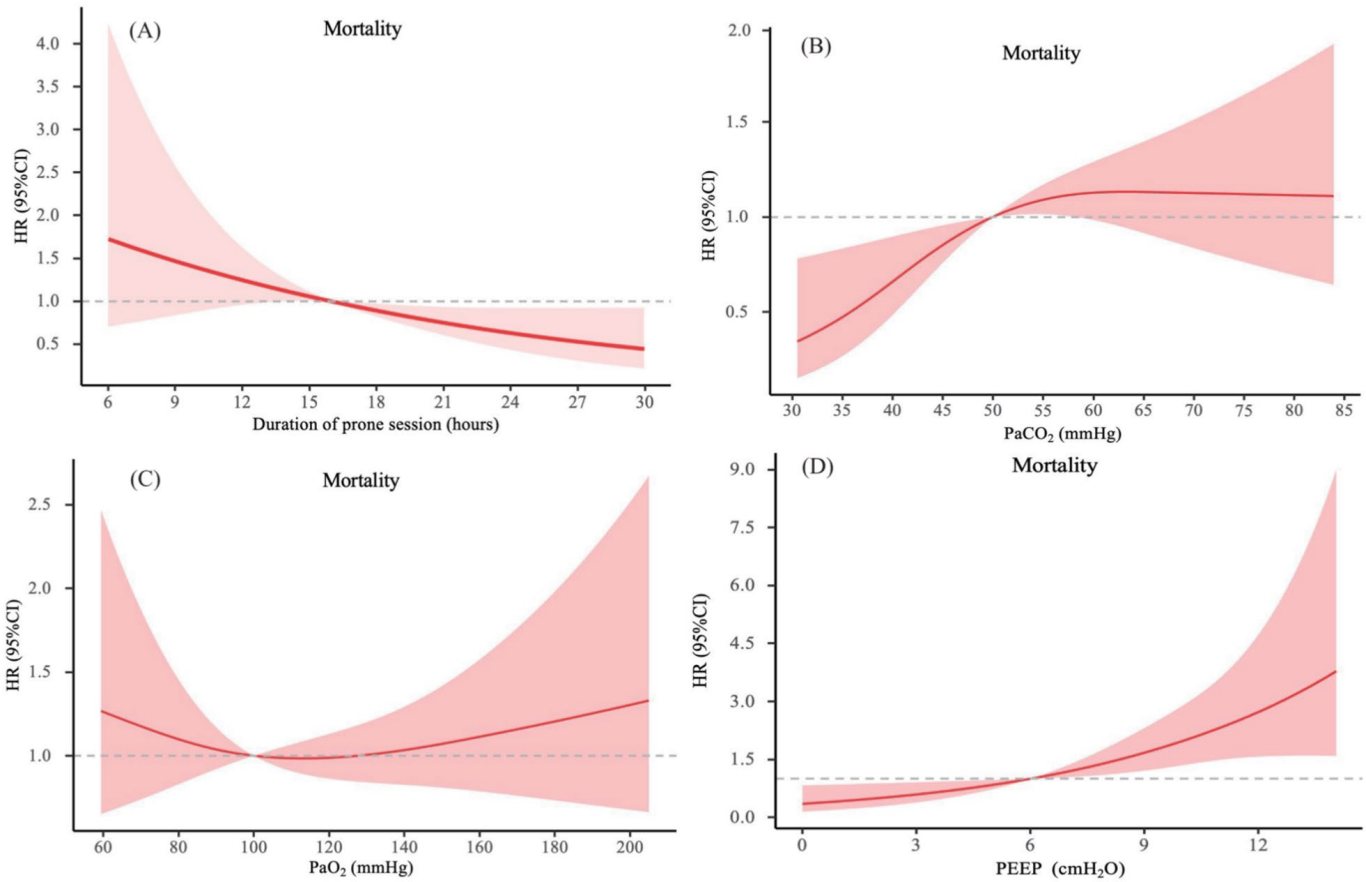
The time–response relationship association between duration of prone session, PaCO_2_, PaO_2_, PEEP and 28-day mortality. Cubic spline curves are shown as a solid line, with the shaded area representing the 95% CI. **A** The time–response relationship association between duration of prone session and 28-day mortality; **B** The time–response relationship association between PaCO_2_ and 28-day mortality; **C** The time–response relationship association between PaO_2_ and 28-day mortality; **D** The time–response relationship association between PEEP and 28-day mortality. *PEEP* positive end-expiratory pressure, *HR* hazard ratio, *CI* confidence interval

**Fig. 4:**
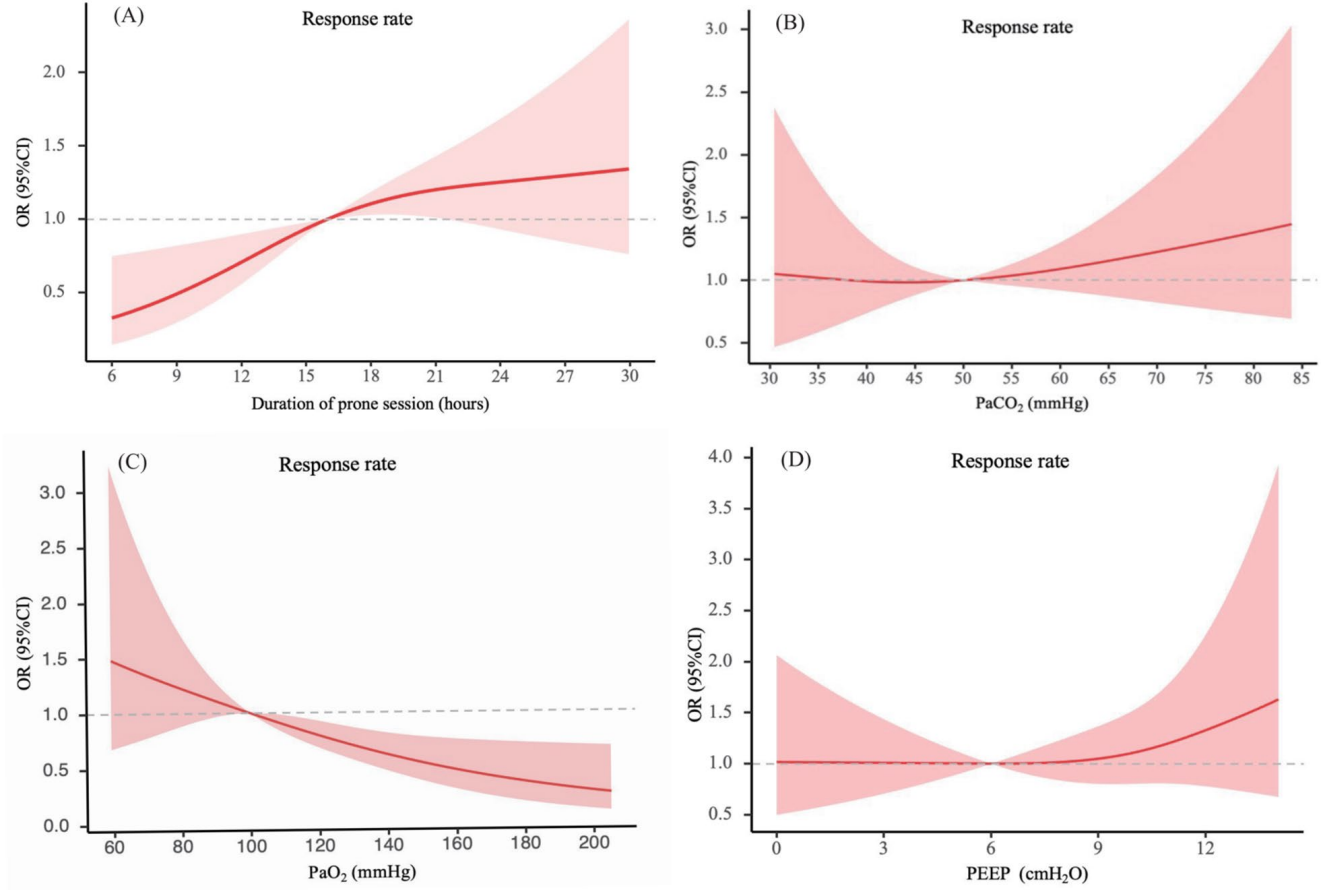
Time–response relationship between duration of prone session, PaCO_2_, PaO_2_, PEEP and response rate. Cubic spline curves are shown as a solid line, with the shaded area representing the 95% CI. **A** Time–response relationship association between duration of prone session and response rate; **B** time–response relationship association between PaCO_2_ and response rate; **C** time–response relationship association between PaO_2_ and response rate; **D** time–response relationship association between PEEP and response rate. *PEEP* positive end-expiratory pressure, *OR* odds ratio, *CI* confidence interval

Figures 3 and 4 and Table S6 show the impact of duration of prone session, PaCO_2_, PaO_2_, PEEP on 28-day mortality and response rate. The results showed that as the duration of prone positioning increased, the risk of mortality significantly decreased, while the risk of response rate increased (Figs. 3A, 4A). As shown in Table S6, PaCO_2_ levels greater than 50 mmHg were associated with to an increased risk of 28-day mortality (HR [95% CI] 1.91 [1.07–3.44], *P* = 0.03) (Fig. 3B), with no benefits observed regarding response rates (Fig. 4B). While no statistical differences were observed in 28-day mortality concerning PaO_2_ level (Fig. 3C), values exceeding 100 mmHg was associated with reduced response rates (OR [95% CI] 0.42 [0.33–0.94], *P* = 0.01) (Fig. 4C). In addition, PEEP values exceeding 10 cmH_2_O were associated with higher 28-day mortality (Fig. 3D).

**Fig. 5:**
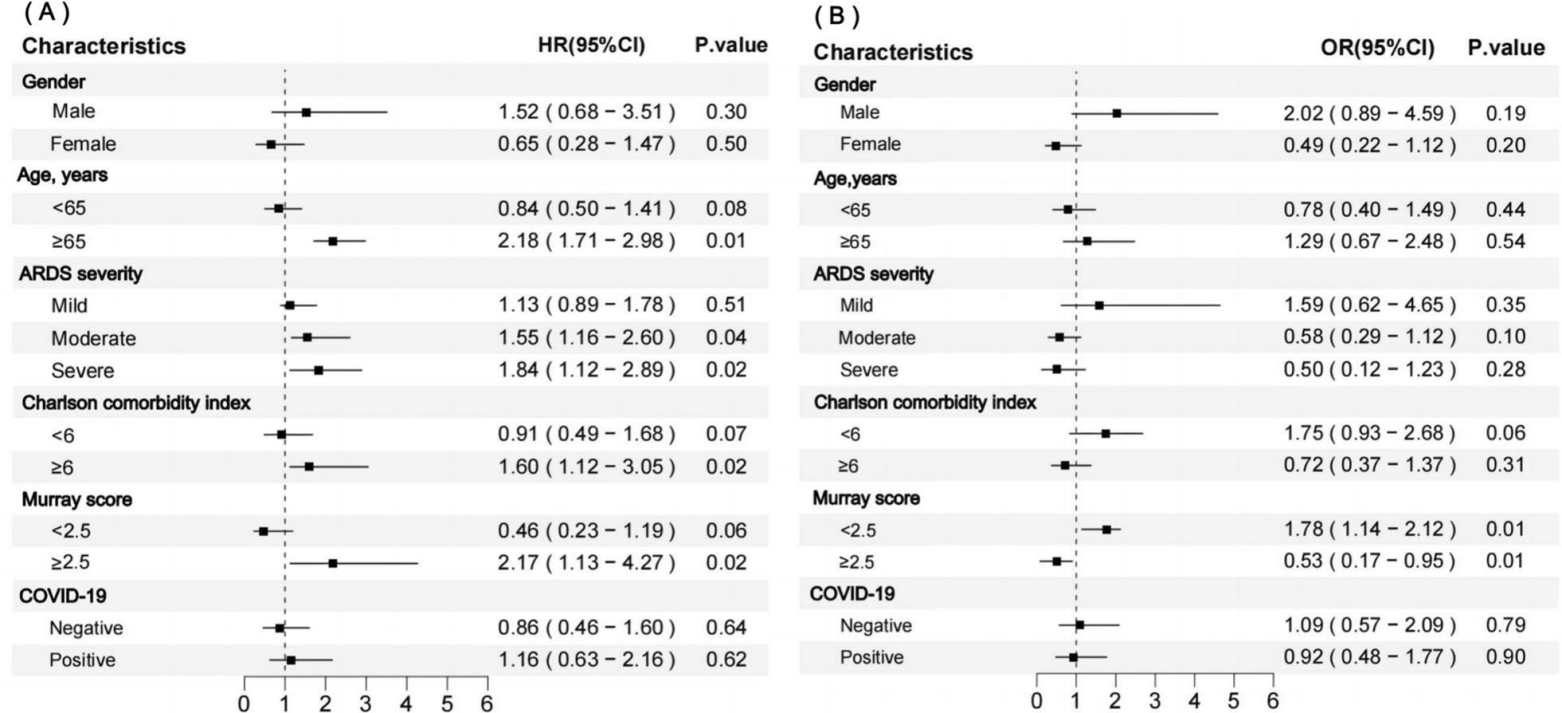
Association between the duration of prone session, 28-day mortality and response rate in subgroups. **A** Association between the duration of prone session and 28-day mortality in subgroups. **B** Association between the duration of prone session and response rate in subgroups. *ARDS* acute respiratory distress syndrome, *OR* odds ratio, *CI* confidence interval

Figure 5, respectively, presents the results of subgroup analyses for 28-day mortality and response rate after PSM. For 28-day mortality, PPP more effectively improved outcomes in patients with moderate to severe ARDS, age ≥ 65, CCI ≥ 6, and a Murray score ≥ 2.5. However, COVID-19 and gender were not associated with the impact of PPP on mortality (Fig. 5A). In terms of response rate, PPP improved patient response regardless of their Murray score (Fig. 5B).

## Discussion

This study enhances the existing strategy for prone positioning duration in ARDS patients by evaluating its impact on both short-term clinical outcomes and physiological responses. Our findings demonstrate that PPP consistently yielded better outcomes, including improved response rates, reduced mortality and improved oxygenation index within specific ranges. For the first time, we systematically analyzed the effects of prone positioning duration on blood gas parameters and ventilatory settings using RCS analysis to identify a time–response relationship. We identified lower PEEP values and reduced levels of PaCO_2_ as potential indicators of improved prognosis. However, no significant differences were found regarding ICU or total hospital stay durations, duration on mechanical ventilation or reintubation rates.

As a lung recruitment technique, prone positioning enhances oxygenation, reduces PaCO₂, and improves right heart function, contributing to lung-protective ventilation strategies [24, 29]. Clinical evidence suggests that prone positioning minimizes overdistension in anterior lung regions and improves posterior alveolar recruitment, promoting optimal compliance and ventilation uniformity [30, 31]. The physiological benefits of PPP stem from reduced stress and strain on the lungs, more uniform alveolar expansion, and stabilized gas exchange [32, 33]. In addition, prolonged sessions may allow delayed physiological benefits to manifest, whereas premature cessation could limit their full potential [33, 34]. A prospective cohort study found that the beneficial physiological effects, such as increased static and dynamic compliance, persists for up to 24 h after prone positioning [34], supporting our findings on improved response rate and survival linked to PPP. In this study, the observed improvement in survival may be attributed to these physiological mechanisms. Further prospective studies should investigate the relationship between prone positioning duration and its physiological effects.

VILI is a critical complication in ARDS management [31, 35], often resulting from repetitive alveolar collapse and reopening during frequent repositioning [22, 36]. In animal studies, PPP may reduce lung injury by enhancing ventilation uniformity, reducing the need for frequent repositioning [6]. In this study, the number of prone position sessions for the PPP group was 2, while for the SPP group, it was 3. This indicates that the PPP group had fewer repositioning sessions. Although we did not assess the impact of PPP on patients' VILI, the better prognosis in patients may be attributed to fewer repositioning sessions and less severe VILI [37, 38]. Whether the benefits stem from improvements in lung compliance or reductions in VILI, PPP offers distinct advantages.

This study suggests that PPP is an effective treatment for ARDS patients with respiratory failure. Subgroup analysis indicates that PPP significantly improves prognosis, particularly among patients with PaO_2_ < 60 mmHg and PaCO_2_ > 50 mmHg. A 2003 study also noted that a decrease in PaCO_2_ following prone positioning can predict survival outcomes in ARDS patients [39], which aligns with our findings. This effect primarily attributed to increased recruitable lung volume, enhanced recruitment of dorsal lung gas, and improved redistribution of blood flow [31, 40]. A small cohort study highlighted that oxygenation improvement significantly begins within 8 h post-prone positioning but does not show further enhancement after 16 h [41]. In contrast, this study demonstrates continued benefits beyond 16 h. This difference may be explained by the smaller sample size (15 patients) and lower severity of lung involvement (PaO_2_/FiO_2_ ratio at 200 mmHg) in the referenced study. Moreover, the PROSEVA study indicated that extended prone positioning for 17 h significantly reduces mortality in severe ARDS patients with PaO_2_/FiO_2_ ratio < 150 mmHg [42]. Similarly, this study observed improved outcomes in the subgroup of patients with moderate to severe ARDS, demonstrating enhanced prognosis with PPP. Consequently, our findings underscore that PPP is particularly beneficial for more severe ARDS cases. In addition, RCS analysis suggest that durations exceeding 24 h may also be feasible, indicating a potential direction for future research.

Although the latest Cochrane evidence-based medicine review did not show a significant survival benefit with high PEEP levels, all clinical guidelines recommend limiting PEEP due to its potential adverse effects [43]. However, no single method for selecting PEEP has proven superior to others. Previous studies have indicated that tailoring PEEP levels based on patient-specific conditions and physiological responses can optimize oxygenation, reduce lung injury, and potentially improve prognosis [44]. In this study, PEEP > 10 cmH_2_O were associated with higher 28-day mortality, indicating that PPP may be needed for patients with difficult lung recruitment. PEEP alone may not adequately promote recruitment in severe ARDS patients and could lead to overdistension of aerated lung parenchyma [45]. PEEP and PPV synergistically improve regional compliance and minimize transpulmonary driving pressure (ΔPTP) in both dependent and non-dependent lung regions [44, 46]. In clinical practice, PEEP primarily aims to enhance lung function by recruiting collapsed alveoli [45]. PEEP settings are personalized based on FiO2, plateau pressure, PaCO_2_, and blood gas analysis [47]. During prone positioning, appropriate adjustments to PEEP levels and ventilator parameters can further facilitate the alveolar recruitment. Future studies should evaluate [36] the fine titration of PEEP after prone positioning to optimize patient outcomes.

Moreover, this study is the first to evaluate the relationship between prone positioning duration and patient response, which defined as an increase of > 20 mmHg in the PaO_2_/FiO_2_ ratio post-prone positioning [18, 23]. The response is a crucial predictor for outcomes and guiding treatment strategies. Prior studies have indicated that improvement in the PaO_2_/FiO_2_ ratio after prone positioning are associated with reduced mortality in COVID-19 patients [48, 49]. This study extends this to ARDS from other causes, demonstrating that improvement in the PaO_2_/FiO_2_ ratio correlate with a reduction in 28-day mortality. PPP facilitate alveolar recruitment and improve gas exchange and subsequently enhance oxygenation. RCS analysis revealed that longer prone durations were associated with a higher response rate, and enhancing prone responsiveness is key to better outcomes.

Safety analysis revealed no significant difference in overall prone-related complications between prolonged and conventional prone positioning groups (50.0% vs. 47.9%), consistent with existing literature (40–60%) [12, 50, 51]. Given the unavoidable missing values, sensitivity analyses were performed to account for missing data using both worst/best-case imputation and multiple imputation methods, while the results were consistent with the main study (Tables S4 and S5). Pressure injuries were the most frequent complication (38.2% overall), with a non-significant trend toward higher incidence in the prolonged group (40.0% vs. 37.0%, *P* = 0.138). Although differences in the number of prone sessions were not statistically significant, single-session differences suggest resource savings for clinical nurses. In resource-constrained ICUs, this reduction in workload is particularly important. This reduction in workload is crucial, as it not only benefits patients but also encourages a reevaluation of prone position frequency, ultimately optimizing both patient outcomes and nursing efficiency.

This study has several limitations that should be considered. First, being a single-center study introduces potential biases, as the majority of patients admitted were critically ill, elderly, or had multiple comorbidities, resulting in a higher expected mortality rate and making it difficult to achieve favorable outcomes with prone ventilation treatment. Second, due to the definition of a non-responsive prone position, this study only analyzed data from the first prone session after ICU admission, potentially overlooking subsequent responses to therapy. Despite efforts such as careful propensity score matching and multivariable analysis, residual confounding factors cannot be completely eliminated. Third, as this was a retrospective study without a standardized protocol, we were unable to track specific criteria for the initiation of prone positioning. All treatment decisions were made by clinical doctors based on individual patient conditions. Although we made effort to collect complication data, there remained some notable missing values. Despite conducting sensitivity analyses to assess the robustness of the results, caution was still needed in interpreting the findings. The future should focus on conducting prospective study to evaluate the impact of prolonged prone positioning strategies on patients and determine the optimal duration of prone ventilation.

## Conclusion

This study evaluated the impact of PPP on the response and outcomes of ARDS patients, demonstrating that prone positioning for ≥ 16 h is associated with significant improvements in short-term clinical indicators and 28-day survival rates. While benefiting patients, this approach may also potentially lead to a reduction in clinical workload for healthcare providers. However, these results require validation through future randomized controlled trials.

## Supplementary Information

Supplementary material 1.

## Data Availability

The data sets used in the present study are available from the first author and corresponding authors on reasonable request.

## Abbreviations

ARDS: Acute respiratory distress syndrome
ICU: Intensive care unit
PPV: Prone positioning ventilation
VILI: Ventilator-induced lung injury
COVID-19: Coronavirus disease 2019
SOFA: Sequential organ failure assessment
APACHE II: Acute physiology and chronic health evaluation II
ECMO: Extracorporeal membrane oxygenation
PPP: Prolonged prone positioning
SPP: Standard prone positioning
MV: Mechanical ventilation
PaO_**2**_: Arterial partial pressure of oxygen
PaCO_**2**_: Arterial partial pressure of carbon dioxide
PEEP: Positive end-expiratory pressure
FiO_**2**_: Fraction inspired of oxygen
HR: Hazard ratio
RR: Relative risk
OR: Odds ratio
CI: Confidence interval
IQR: Interquartile ranges
PSM: Propensity score matching
RCS: Restricted cubic splines
SMD: Standardized mean difference
