# Supplementary material for: The effects of prolonged prone positioning on response and prognosis in patients with acute respiratory distress syndrome: a retrospective cohort study
Source: J Intensive Care. 2025 May 7;13:24. doi: 10.1186/s40560-025-00795-x (PMC12057286; doi:10.1186/s40560-025-00795-x)

**Supplementary Material**

**Table S1: Baseline characteristics between two groups after propensity score matching.**

**Table S2. Prone information and respiratory parameters before and after proning.**

**Table S3:** **Missing number (%) for prone positioning-related complications.**

**Table S4. Sensitivity analysis results of the worst-case imputation and best-case imputation methods for missing data**.

**Table S5. Sensitivity analysis results of multiple imputation methods for missing data.**

**Table S6: The time-response relationship between response rate, and 28 day mortality.**

**Figure S1: Standardized mean difference (SMD) of variables before and after propensity score matching.**

**Figure S2: Common support domain plot after propensity score matching.**

**Figure S3: Jitter plot of distributions before and after propensity score matching.**

**Figure S4. Density curve of prone ventilation complications before and after multiple interpolation.**

**Table S1. Baseline characteristics between two groups after PSM.** After PSM, the baseline profiles were well balanced between the two groups with SMDs <10% for all variables

| Items | Overall (n=162) | SPP group(n=81) | PPP group(n=81) | *P* | SMD |
| --- | --- | --- | --- | --- | --- |
| Age | 63.00 [52.00, 76.00] | 63.00 [51.00, 72.00] | 64.00 [55.00, 78.00] | 0.145 | 0.065 |
| Gender [*n*(%)] |  |  |  | 0.587 | 0.072 |
| Male | 132 (81.5) | 67 ( 82.7) | 65 ( 80.2) |  |  |
| Female | 30 (17.9) | 14 ( 17.3) | 16 ( 19.8) |  |  |
| Height (m) | 160.0 [155.0, 170.0] | 160.0 [154.0, 168.0] | 160.0 [155.0, 170.0] | 0.338 | 0.031 |
| Weight (kg) | 60.9 [50.1, 66.2] | 60.0 [51.0, 65.0] | 61.0 [50.5, 66.0] | 0.138 | 0.002 |
| Body mass index (kg/m^2^) | 23.0 [21.0, 24.9] | 22.9 [20.9, 25.0] | 23.0 [21.1, 24.6] | 0.881 | 0.031 |
| Etiologies [*n*(%)] |  |  |  |  |  |
| Pneumonia | 160 (98.8) | 79 ( 97.5) | 81 (100.0) | 0.477 | 0.051 |
| Sepsis | 58 (35.8) | 28 ( 34.6) | 30 ( 37.0) | 0.87 | 0.034 |
| Trauma | 9 ( 5.6) | 4 ( 4.9) | 5 ( 6.2) | 1 | 0.039 |
| Other etiologies | 13 ( 8.0) | 8 ( 9.9) | 5 ( 6.2) | 0.563 | 0.029 |
| ARDS severity [*n*(%)] |  |  |  | 0.973 | 0.021 |
| Mild | 23 (14.2) | 12 ( 14.8) | 11 ( 13.6) |  |  |
| Moderate | 93 (57.4) | 46 ( 56.8) | 47 ( 58.0) |  |  |
| Severe | 46 (28.4) | 23 ( 28.4) | 23 ( 28.4) |  |  |
| Microbiology |  |  |  |  |  |
| Virus [*n*(%)] | 136 (84.0) | 68 ( 84.0) | 68 ( 84.0) | 1 | 0.001 |
| Bacteria [*n*(%)] | 124 (76.5) | 62 ( 76.5) | 62 ( 76.5) | 1 | 0.001 |
| Fungus [*n*(%)] | 72 (44.4) | 34 ( 42.0) | 38 ( 46.9) | 0.635 | 0.001 |
| APACHE II | 24.00 [19.00, 29.00] | 23.00 [20.00, 28.00] | 24.00 [18.00, 31.00] | 0.68 | 0.089 |
| SOFA score | 8 [5, 1] | 8 [5, 10] | 8 [5, 10] | 0.902 | 0.008 |
| Murrary score | 3.00 [2.50, 3.50] | 3.00 [2.00, 3.50] | 3.00 [2.50, 3.50] | 0.603 | 0.030 |
| Charlson index | 6 [4, 7] | 5 [4, 7] | 6 [4, 7] | 0.833 | 0.001 |
| Number of the prone position sessions | 2 [1, 5] | 3 [1, 4] | 2 [1, 5] | 0.129 | 0.009 |
| Days from ICU to pronation (day) | 1.44 [0.72, 4.88] | 1.60 [0.87, 5.96] | 1.29 [0.38, 4.00] | 0.126 | 0.045 |
| Treatment [*n*(%)] | | | | | |
| Mechanical Ventilation | 151 (93.2) | 74 ( 91.4) | 77 ( 95.1) | 0.063 | 0.093 |
| Muscle relaxants | 63 ( 38.9) | 30 ( 37.0) | 33 ( 40.7) | 0.098 | 0.042 |
| ECMO | 22 (13.6) | 12 ( 14.8) | 10 ( 12.3) | 0.124 | 0.029 |
| Mechanical ventilation parameters | | | | | |
| PEEP (cmH_2_O) | 10 [8, 10] | 10 [8, 12] | 10 [8, 10] | 0.163 | 0.031 |
| Compliance (mL/cm H_2_O) | 32 [23, 39] | 32 [25, 42] | 31 [23, 37] | 0.307 | 0.002 |
| etCO_2_ (mmHg) | 36 [33, 46] | 37 [33, 43] | 36 [33, 46] | 0.211 | 0.013 |
| FiO_2_ (%) | 70 [50, 100] | 70 [50, 100] | 70 [55, 100] | 0.911 | 0.012 |
| Tidal volume (mL/kg) | 7.1 [6.0, 8.8] | 7.0 [6.0, 9.0] | 7.2 [6.1, 9.0] | 0.731 | 0.004 |
| Arterial blood gas measurements |  |  |  |  |  |
| PaO_2_ (mmHg) | 93.05 [77.08, 130.00] | 95.40 [79.40, 131.00] | 91.80 [75.20, 122.00] | 0.33 | 0.067 |
| PaCO_2_ (mmHg) | 42.80 [37.82, 53.75] | 42.90 [38.00, 48.70] | 42.30 [37.80, 56.70] | 0.675 | 0.045 |
| PaO_2_/FiO_2_ ratio | 149.00 [98.08, 208.50] | 149.00 [99.50, 216.00] | 149.00 [96.00, 200.00] | 0.605 | 0.035 |
| Abbreviations: PSM, propensity score matching; ARDS, acute respiratory distress syndrome; APACHE II, acute physiology and chronic health evaluation II; SOFA, sequential organ failure assessment; ICU, intensive care unit; ECMO, extracorporeal membrane oxygenation; PEEP, positive end-expiratory pressure. | | | | | |

**Table S2. Prone information and respiratory parameters before and after proning**

|  | **Overall (n=234)** | **SPP group (n=133)** | **PPP group (n=101)** | ***P*** |
| --- | --- | --- | --- | --- |
| Prone information |  |  |  |  |
| Number of the prone position sessions | 2 [1, 5] | 3 [1, 5] | 2 [2, 5] | 0.078 |
| Days from ICU to pronation (days) | 1.67 [0.74, 4.70] | 1.75 [0.82, 4.95] | 1.44 [0.69, 4.01] | 0.052 |
| First session prone duration (hours) | 14.91 [11.50, 17.87] | 11.00 [8.13, 14.12] | 20.13 [17.82, 25.60] | 0.001 |
| Total duration of prone in ICU (hours) | 40.13 [19.31, 85.90] | 30.91 [10.31, 40.52] | 58.51 [23.19. 80.13] | 0.001 |
| Days on which prone was performed (days)^a^ | 3 [2, 6] | 3 [2, 5] | 3 [2, 6] | 0.173 |
| Respiratory parameters |  |  |  |  |
| PEEP (cmH_2_O) |  |  |  |  |
| before prone | 10 [8, 10] | 10 [8, 11] | 10 [8, 10] | 0.703 |
| after prone | 9 [7, 10] | 10 [8, 10] | 10 [8, 10] | 0.118 |
| Compliance (mL/cm H_2_O) |  |  |  |  |
| before prone | 32 [23, 40] | 32 [22, 40] | 32 [24, 39] | 0.773 |
| after prone | 31 [20, 40] | 31 [23, 39] | 32 [25, 40] | 0.421 |
| etCO_2_ (mmHg) |  |  |  |  |
| before prone | 35 [33, 47] | 37 [33, 46] | 36 [34, 46] | 0.182 |
| after prone | 34 [35, 50] | 35 [33, 49] | 35 [35, 50] | 0.081 |
| FiO_2_ (%) |  |  |  |  |
| before prone | 70 [50, 100] | 70 [50, 100] | 70 [55, 100] | 0.711 |
| after prone | 60 [45, 90] | 60 [45, 90] | 60 [40, 85] | 0.499 |
| Tidal volume (mL/kg) |  |  |  |  |
| before prone | 7.3 [6.0, 9.0] | 7.1 [5.9,9.1] | 7.4 [6.1,9.0] | 0.314 |
| after prone | 6.9 [5.6, 8.8] | 6.8 [5.9,8.5] | 7.1 [5.9,8.5] | 0.181 |
| Abbreviations: ICU, intensive care unit; PEEP, positive end-expiratory pressure.  ^a^ If the patient was performed in the prone position in a day, that day was recorded as a prone position day. | | | | |

**Table S3. The time-response relationship between response rate, and 28 day mortality.**

|  | | | 28 day mortality | | | Response rate | | |
| --- | --- | --- | --- | --- | --- | --- | --- | --- |
|  | HR | 95%CI | | *P* | OR | | 95%CI | *P* |
| Duration of prone session (h) |  |  | |  |  | |  |  |
| ＜16 | 1.00 |  | |  | 1.00 | |  |  |
| 16-24 | 0.66 | 0.23 - 0.89 | | 0.03 | 1.12 | | 1.09 - 1.73 | 0.01 |
| ＞24 | 0.51 | 0.35 - 0.74 | | 0.01 | 1.54 | | 0.81 - 2.31 | 0.09 |
| PaO_2_ (mmHg) |  |  | |  |  | |  |  |
| ＜60 | 1.00 |  | |  | 1.00 | |  |  |
| 60-100 | 1.21 | 0.87 - 1.45 | | 0.81 | 1.24 | | 0.68 - 2.01 | 0.51 |
| ＞100 | 1.08 | 0.79 - 1.33 | | 0.14 | 0.42 | | 0.33 - 0.94 | 0.01 |
| PaCO_2_ (mmHg) |  |  | |  |  | |  |  |
| ≤50 | 1.00 |  | |  | 1.00 | |  |  |
| ＞50 | 1.91 | 1.07 - 3.44 | | 0.03 | 1.16 | | 0.63 - 2.15 | 0.63 |
| PEEP (cmH_2_O) |  |  | |  |  | |  |  |
| ＜6 | 1.00 |  | |  |  | |  |  |
| 6 - 10 | 1.43 | 0.93 - 1.97 | | 0.09 | 1.03 | | 0.66 - 1.48 | 0.13 |
| ＞10 | 1.89 | 1.12 - 5.89 | | 0.01 | 1.19 | | 0.79 - 2.45 | 0.76 |
| Abbreviations: HR, hazard ratio; OR, odds ratio; CI, confidence interval; PEEP, positive end-expiratory pressure. | | | | | | | | |

| **Prone positioning-related complications** | **All missing count (%)**  **Overall (n=234)** | **SPP group**  **(n=133)** | **PPP group**  **(n=101)** | ***P*** |
| --- | --- | --- | --- | --- |
| Any complication of proning, n(%) | 111 ( 47.4) | 60 (45.1) | 51 (50.5) | 0.128 |
| Catheter-related events, n(%) | 111 ( 47.4) | 60 (45.1) | 51 (50.5) | 0.128 |
| Hemodynamic instability, n(%) | 111 ( 47.4) | 60 (45.1) | 51 (50.5) | 0.128 |
| Pressure injury, n(%) | 111 ( 47.4) | 60 (45.1) | 51 (50.5) | 0.128 |
| Gastrointestinal complications, n(%) | 111 ( 47.4) | 60 (45.1) | 51 (50.5) | 0.128 |
| Other complications, n(%) | 111 ( 47.4) | 60 (45.1) | 51 (50.5) | 0.128 |

**Table S4.** **Missing number (%) for prone positioning-related complications.**

**Table S5. Sensitivity analysis results of the worst-case imputation and best-case imputation methods for missing data**

| **Prone positioning-related complications** | **Overall (n=234)** | **SPP group (n=133)** | **PPP group (n=101)** | ***P*** |
| --- | --- | --- | --- | --- |
| **Worst-case Imputation** |  |  |  |  |
| Any complication of proning, n(%) | 171 (73.1) | 95 (71.4) | 76 (75.2) | 0.061 |
| Catheter-related events, n(%) | 114 (48.7) | 62 (46.6) | 52 (50.5) | 0.181 |
| Hemodynamic instability, n(%) | 124 (53.0) | 67 (50.4) | 56 (55.4) | 0.077 |
| Pressure injury, n(%) | 158 (67.5) | 87 (65.4) | 71 (70.3) | 0.138 |
| Gastrointestinal complications, n(%) | 128 (54.7) | 70 (52.6) | 58 (57.4) | 0.054 |
| Other complications, n(%) | 124 (53.0) | 68 (51.1) | 56 (55.4) | 0.114 |
| **Best-case Imputation** |  |  |  |  |
| Any complication of proning, n(%) | 60 (25.6) | 35 (26.3) | 25 (24.8) | 0.101 |
| Catheter-related events, n(%) | 3 ( 1.3) | 2 ( 1.5) | 1 ( 1.0) | 0.889 |
| Hemodynamic instability, n(%) | 13 ( 5.6) | 7 ( 5.3) | 5 ( 5.0) | 0.783 |
| Pressure injury, n(%) | 47 (20.1) | 27 (20.3) | 20 (19.8) | 0.476 |
| Gastrointestinal complications, n(%) | 17 ( 7.3) | 10 ( 7.5) | 7 ( 6.9) | 0.944 |
| Other complications, n(%) | 13 ( 5.6) | 8 ( 6.0) | 5 ( 5.0) | 0.167 |

**Table S6. Results of complications related to prone position after multiple interpolation.**

| **Prone positioning-related complications** | **Overall (n=234)** | **SPP group (n=133)** | **PPP group (n=101)** | ***P*** |
| --- | --- | --- | --- | --- |
| Any complication of proning, n(%) | 115 (49.1) | 64 (48.1) | 51 (50.5) | 0.134 |
| Catheter-related events, n(%) | 5 ( 2.1) | 3 ( 2.3) | 2 ( 2.0) | 0.672 |
| Hemodynamic instability, n(%) | 24 (10.2) | 13 (9.8) | 11 (10.9) | 0.838 |
| Pressure injury, n(%) | 91 (38.9) | 50 (37.6) | 42 ( 41.6) | 0.059 |
| Gastrointestinal complications, n(%) | 33 (14.1) | 19 (14.3) | 14 (13.9) | 0.324 |
| Other complications, n(%) | 24 (10.3) | 14 (10.5) | 10 ( 9.9) | 0.117 |

**Figure S1. Standardized mean difference (SMD) of variables before and after propensity score matching.** After matching, the baseline profiles were well balanced between the two groups with SMDs <10% for all variables.

Abbreviations: CRRT, continuous renal replacement therapy; MV, mechanical ventilation; ARDS, acute respiratory distress syndrome; APACHE II, acute physiology and chronic health evaluation II; SOFA, sequential organ failure assessment; PPV, prone positioning ventilation.


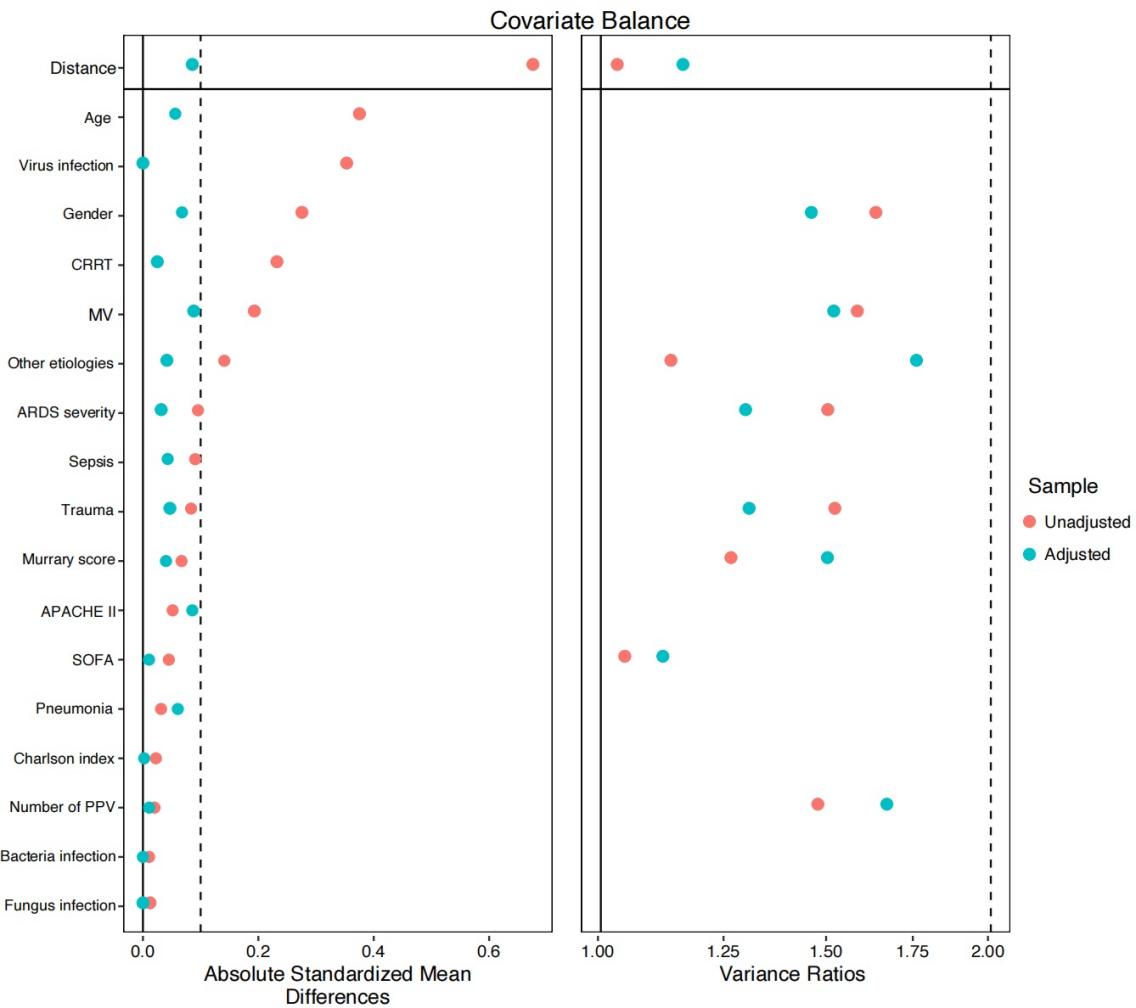


**Figure S2. Common support domain plot after propensity score matching.**


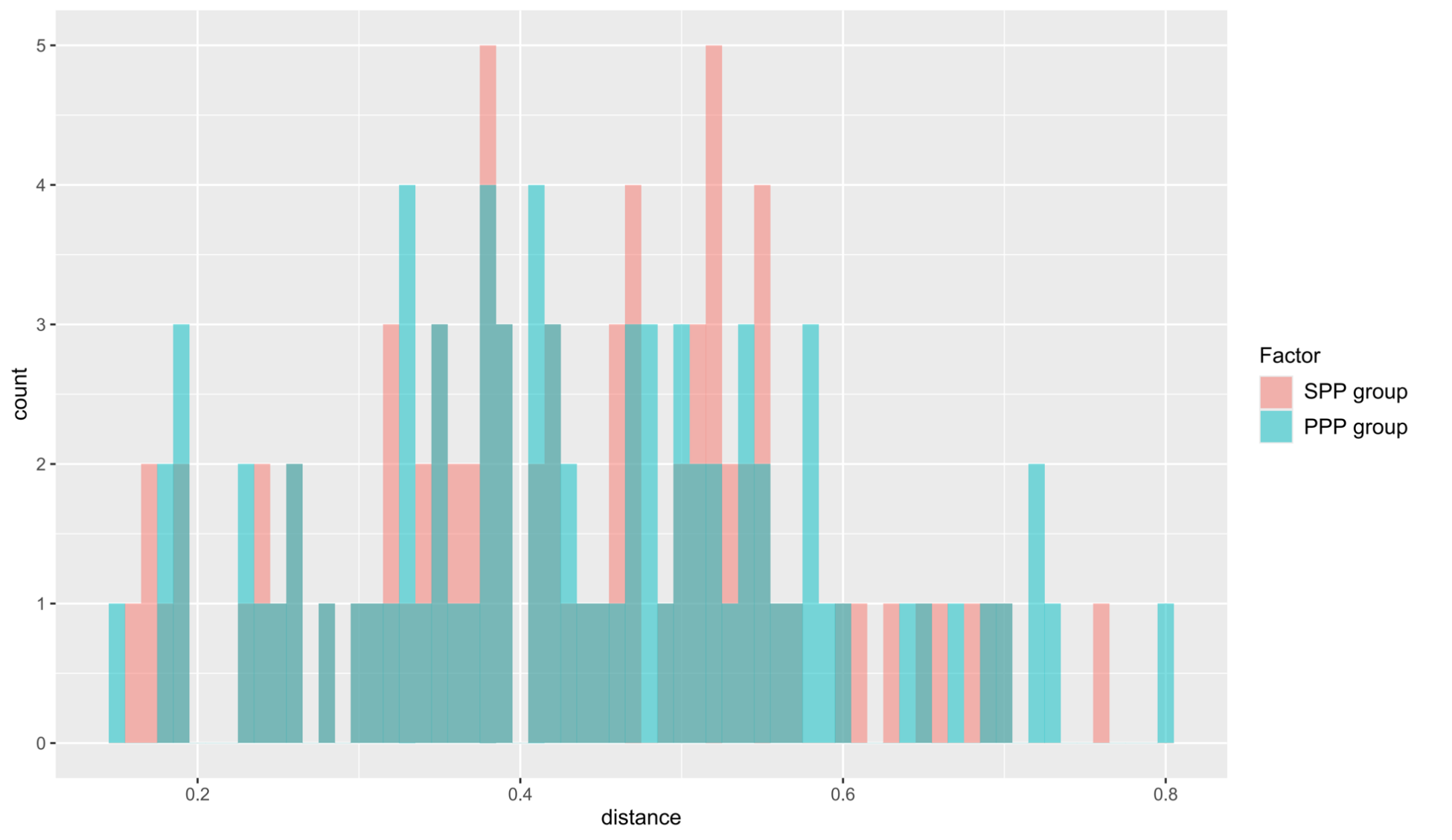


**Figure S3. Jitter plot of distributions before and after propensity score matching.**


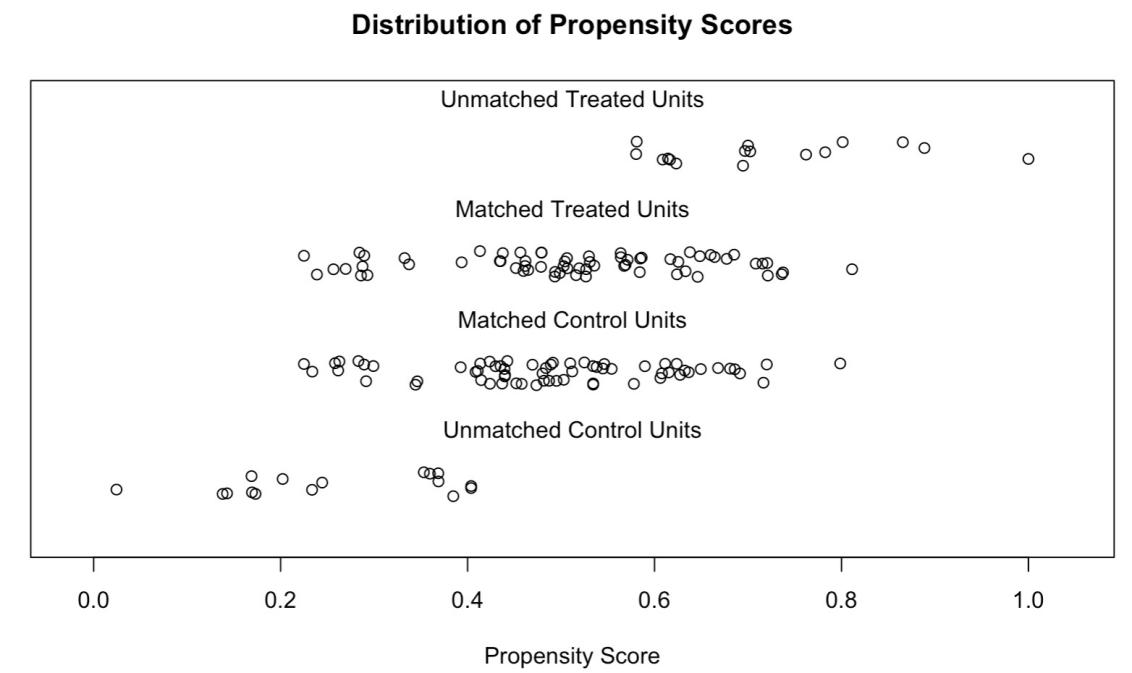


**Figure S4. Density curve of prone ventilation complications before and after multiple interpolation.** The black curve represents the original data, while the red curve represents the results after five imputations. A high degree of overlap indicates a good imputation effect.


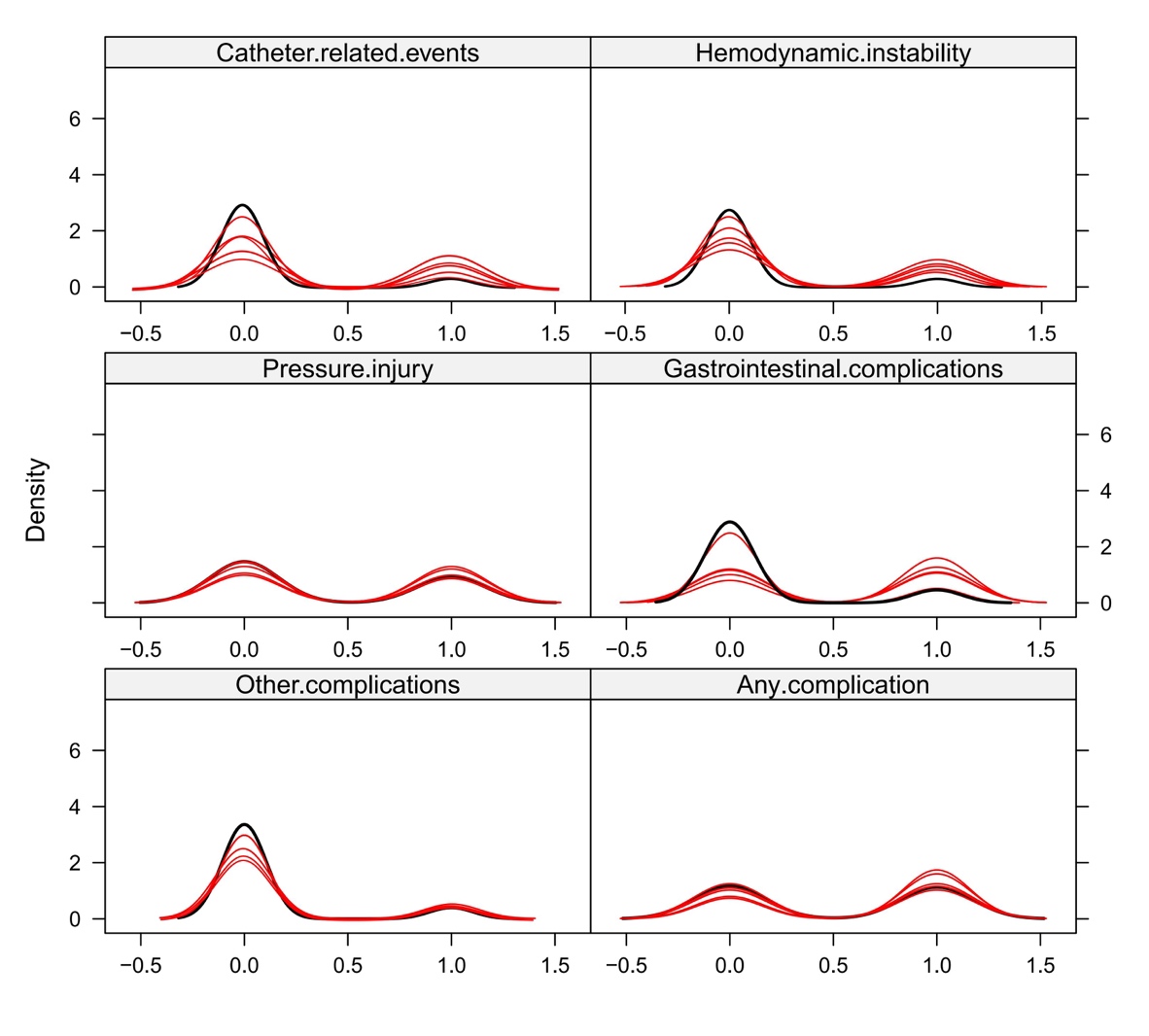

Supplement: Supplementary file 1 — Supplementary material 1. [file 40560_2025_795_MOESM1_ESM.docx]
